# Ethanol Solvation of Polymer Residues in Graphene Solution-Gated Field Effect Transistors

**DOI:** 10.1021/acssuschemeng.4c01538

**Published:** 2024-06-04

**Authors:** Juan Pedro Merino, Sergi Brosel-Oliu, Gemma Rius, Xavi Illa, Manuel Vázquez Sulleiro, Elena Del Corro, Eduard Masvidal-Codina, Andrea Bonaccini Calia, Jose Antonio Garrido, Rosa Villa, Anton Guimerà-Brunet, Maurizio Prato, Alejandro Criado, Elisabet Prats-Alfonso

**Affiliations:** † Center for Cooperative Research in Biomaterials (CIC biomaGUNE), Basque Research and Technology Alliance (BRTA), Paseo de Miramon 194, Donostia-San Sebastián 20014, Spain; ‡ Institute of Microelectronics of Barcelona (IMB-CNM, CSIC), Campus UAB, Bellaterra 08193, Spain; § Centro de Investigación Biomédica en Red de Bioingeniería, Biomateriales y Nanomedicina, Instituto de Salud Carlos III, Madrid 28029, Spain; ∥ Department of Chemical and Pharmaceutical Sciences, University of Trieste, Via L. Giorgieri 1, Trieste 34127, Italy; ⊥ Universidade da Coruña, CICA − Centro Interdisciplinar de Química e Bioloxía, Rúa as Carballeiras, A Coruña 15071, Spain; # Catalan Institute of Nanoscience and Nanotechnology (ICN2), CSIC and BIST, Campus UAB, Bellaterra, Barcelona 08193, Spain; ∇ ICREA Pg, Barcelona 08010, Spain; ○ Ikerbasque, Basque Foundation for Science, Bilbao 48013, Spain

**Keywords:** graphene, polymer residues, ethanol, green solvent, solution-gated field-effect transistors (SGFETs)

## Abstract

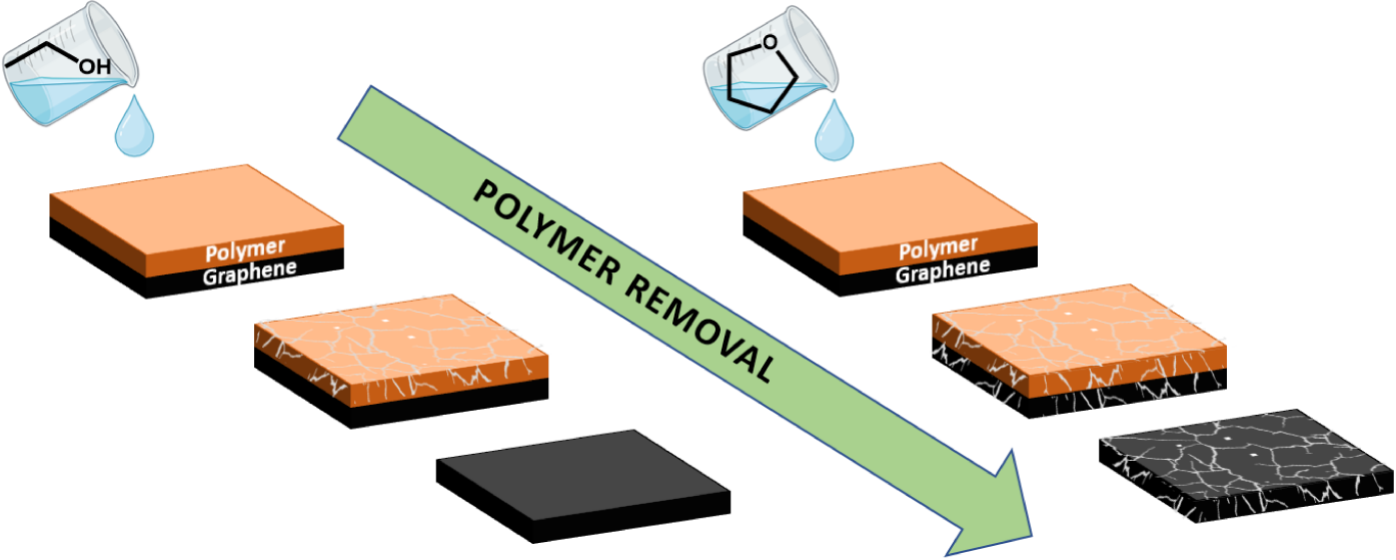

The persistence of photoresist residues from microfabrication procedures causes significant obstacles in the technological advancement of graphene-based electronic devices. These residues induce undesired chemical doping effects, diminish carrier mobility, and deteriorate the signal-to-noise ratio, making them critical in certain contexts, including sensing and electrical recording applications. In graphene solution-gated field-effect transistors (gSGFETs), the presence of polymer contaminants makes it difficult to perform precise electrical measurements, introducing response variability and calibration challenges. Given the absence of viable short to midterm alternatives to polymer-intensive microfabrication techniques, a postpatterning treatment involving THF and ethanol solvents was evaluated, with ethanol being the most effective, environmentally sustainable, and safe method for residue removal. Employing a comprehensive analysis with XPS, AFM, and Raman spectroscopy, together with electrical characterization, we investigated the influence of residual polymers on graphene surface properties and transistor functionality. Ethanol treatment exhibited a pronounced enhancement in gSGFET performance, as evidenced by a shift in the charge neutrality point and reduced dispersion. This systematic cleaning methodology holds the potential to improve the reproducibility and precision in the manufacturing of graphene devices. Particularly, by using ethanol for residue removal, we align our methodology with the principles of green chemistry, minimizing environmental impact while advancing diverse graphene technology applications.

## Abstract 2

Ethanol exhibits notable efficacy by solvating polymer residues in postprocessed graphene devices by reducing the dispersion of main electrical parameters.

## Introduction

Microelectronic processing routinely employs polymers, e.g., thin film resists in each of the photolithography steps of the microfabrication sequence. In the case of producing sensor devices, photoactive polymers used in photolithography can be the source of organic contamination in the sensing layer.^1^ Generally, when sensor structural materials such as metals or silicon oxide layers are used, the standardized cleaning steps, comprising both chemical and physical techniques, efficiently remove polymer residues. These steps only become critical in the final scaling of silicon nanoelectronics.^2^

However, the incorporation of graphene as a top sensing layer has brought numerous challenges in its integration into microfabrication processes.^3−6^ A frequent issue arises from graphene’s high sensitivity to the absorption and retention of polymeric residues, in contrast to many other materials typically employed in standard microfabrication processes.^2^ Importantly, organic residues negatively alter the electrical performance of graphene devices, such as the signal-to-noise ratio in sensing applications.^7,8^

In the domain of field effect transistors (FETs), these residues play a crucial role, affecting the modulation of electrical current in the graphene channel and the variability in the current-to-voltage curves. In the context of solution-gated field-effect transistors (SGFETs), this lack of homogeneity negatively alters the calibration of these systems, a crucial requirement for sensing and biosensing applications.^9−11^ Specifically, in such applications utilizing graphene-based SGFETs (gSGFET), monitoring changes in charges on the channel during functionalization or detection becomes critical. This emphasizes the importance of controlling contaminants or residues on graphene surfaces. Therefore, it is necessary to assess effective procedures for removing surface contamination from graphene, particularly at the ultimate stage of fabrication, and to consolidate residue removal steps that are compatible with the microfabrication processing and device materials involved.

In this regard, the literature has already detailed several strategies for cleaning graphene. A well-known example is the mechanical cleaning using tip scanning in atomic force microscopy (AFM).^12^ Although this methodology is very effective, its cost and lack of feasibility are due to scalability limitations for large areas or at the wafer scale within a time-efficient framework.

Likewise, another approach poses challenges for scaling up, rendering it irrelevant industrially or applicable only to specific device configurations. This involves current-induced desorption of polymer residues, a method limited to single-device application.^13^ In contrast, other common and scalable approaches involve exposure to ozone^14^ or plasma^15^ environments. They are indeed very effective at cleaning polymers from semiconductor surfaces, but they can damage structurally exposed graphene.^16^ In such cases, it is often very challenging or impractical to adjust ozone exposure conditions for unknown thickness or an inhomogeneous amount of polymer residues. In brief, even after successfully eliminating the polymer, slightly extended exposure to ozone can lead to its penetration to the graphene layer.^17^ This could result in damage to its crystal structure, inducing vacancies and creating oxidation sites locally or across broader areas.

Additional procedures, such as high-temperature annealing and electrostatic force, have also been demonstrated to be effective in removing polymer residues.^18,19^ However, they often tend to be too aggressive, compromising the preservation of graphene surface properties and device performance, and are not always feasible for application at the wafer scale.

Alternatively, a solvent-based methodology is a plausible option due to its simplicity, compatibility with microprocessing, scalability, and cost efficiency. Solvents such as dimethylacetamide^20^ or dioxolane^21^ have been developed and tested effectively on graphene. Their effectiveness is focused on the increase in the electronic mobility when applied to backgated graphene transistors. This improvement is widely demonstrated with reduced polymeric residues.^22,23^ However, the adoption of harsh solvents is unlikely due to their high toxicity to health and the environment.^21^ Other similar solvents have been tested for removing residues from graphene surfaces. In fact, several chemicals are routinely employed in certain intermediate steps of the microprocessing sequences as polymer removers for other materials. However, they cannot always be applied to the graphene layer without causing damage.^24^ These standard chemicals for microelectronics can be divided as (i) solvents, (ii) alkaline media based, and (iii) strippers. In the case of middle-sized molecule solvents, such as a combination of acetone and 2-propanol, they are commonly used but are generally not recommended. This is because acetone often leaves behind organic residues, and 2-propanol may not always penetrate sufficiently to remove them all. Other middle-sized solvents such as *N*-methylpyrrolidone (NMP) are much more effective, but they are not fully compatible with certain polymer substrates, as well as some polymeric passivation layers. Usually, the exposure time of these solvents is not very long, but it is sufficient to interfere with the swelling of the structure substrate and alter its integrity. Therefore, the lack of complete compatibility can be considered to be an issue, making it preferable to seek a safer alternative. Furthermore, the literature describes their inevitable physical adsorption^25^ on the graphene surface, which can alter its physicochemical properties. Likewise, dimethyl sulfoxide (DMSO), along with other strippers based on tetramethylammonium hydroxide (TMAH) and glycerol, is more commonly used. However, they can affect the bonding strength between the deposited graphene and its substrate, potentially reducing graphene adhesion over time and hence promoting delamination.

The gSGFETs also require an additional polymeric layer as passivation, i.e., to expose graphene while isolating and blocking response from the metal contacts, as the measurements are performed in solution. Its patterning process may also leave residues after resist development. To clean surfaces, some organic solvents, as previously mentioned, may have limited utility as they tend to detach this polymer passivation layer.^26^

In this regard, wet chemistry exhibits remarkable versatility, with numerous variations available and applicable to a wide range of electronic devices and configurations. Moreover, it is less likely to induce physical alterations, such as atomic vacancies or stress, compared to other methods.^27^ Nevertheless, selecting the most appropriate chemical for dissolving different polymer layers without compromising graphene structure or causing swelling of the polymeric substrate or passivation entails specific requirements. Miller-Chou et al. observed a decrease in the dissolution rate of polymer chains as the size of solvent molecules increased, suggesting that the rate of dissolution is constrained by the speed at which solvent molecules can penetrate the polymer.^28^ In light of this observation, one might consider using methanol (MeOH) due to its simplicity and low molecular weight. However, it has been documented that certain polymer films can develop cracks when exposed to MeOH, even at relatively low temperatures.^29^ This could potentially compromise the graphene integrity.^28,30^

Instead of using bulky solvents that cannot penetrate the polymer layer, leaving behind numerous residues or polymers that are not fully compatible with substrate polymers, and small solvents that could cause cracks in the residue polymer due to the solvation process, potentially damaging graphene,^28^ ethanol (EtOH) was deliberately chosen to remove the polymeric residues. Moreover, EtOH has low molecular weight, is volatile and easy to remove, and is preferred for industrial applications as it is nontoxic and more environmentally friendly.^31^ Additionally, tetrahydrofuran (THF) was assessed as a potential solvent for polymer residue removal, since it is recognized as one of the most efficient and versatile midsize solvents, with the aim of investigating its effect on graphene layers and the structural polymers constituting the FET device. Although THF is not classified as a green solvent like EtOH, its performance will be assessed in comparison.

Herein, we will analyze the presence of polymer residues, assess the efficacy of solvent removal through polymer solvation, and evaluate the quality and integrity of graphene films in FET structures, specifically focusing on gSGFET for its potential applications in biosensing. Three complementary surface characterization methods, X-ray photoelectron spectroscopy (XPS), AFM, and Raman spectroscopy, will be performed on macrotransistors previously patterned with gold contacts. These transistors will be immersed in EtOH and THF solvents for varying durations. In addition, the electrical characterization of gSGFETs, with transfer curves serving as figures of merit, will be conducted subjected to the same polymer solvation procedures. By combining morphological and electrical studies, we aim to obtain comprehensive data on the reduction of the polymer residues and, particularly, the impact of the passivation layer on these devices.

## Experimental Details

### Reagents

EtOH (Sigma-Aldrich, St. Louis, MO, USA, 99.9%), THF (Sigma-Aldrich, 98%), and phosphate-buffered saline (PBS), 10 mM sodium phosphate, 137 mM sodium chloride, 2.7 mM potassium chloride, pH 7.4 (Sigma-Aldrich, St. Louis, MO, USA), 2-propanol (Microchemicals, Germany), poly(methyl methacrylate) (PMMA) (Kayaku Advanced Materials, Westborough, MA, USA), AZ5214E (Clariant, Germany), and SU-8 (Microchem, Germany) were used.

### Graphene Growth, Transfer, and Device Fabrication

Graphene materials were grown by the chemical vapor deposition (CVD) method. A lamp-heated rapid thermal CVD equipment from Jipelec was employed, and substrates consisted of 25 μm thick, 99.8% metal basis copper foils provided by Alfa Aesar. Prior to the CVD process, copper foils had been cut into 6 cm × 5 cm samples, sequentially cleaned in acetic acid and acetone, and finally rinsed in isopropyl alcohol (IPA). Graphene growth processing sequence and conditions included a first step of Cu annealing at 750 °C, for 10 min, in a 200 sccm H_2_ flow, followed by graphene deposition at 800 °C, for 5 min, in a 25:200 sccm CH_4_:H_2_ flow. CVD graphene was delaminated from the Cu foil by the electrochemical method^32^ and using as transfer media, a 700 nm thick poly(methyl methacrylate) (PMMA) film (950k MW dissolved in anisole) deposited by spin coating. Once transferred to the target surface, the PMMA protection layer is removed in acetone and isopropanol by 30 min immersion in each solvent.

**Figure 1:**
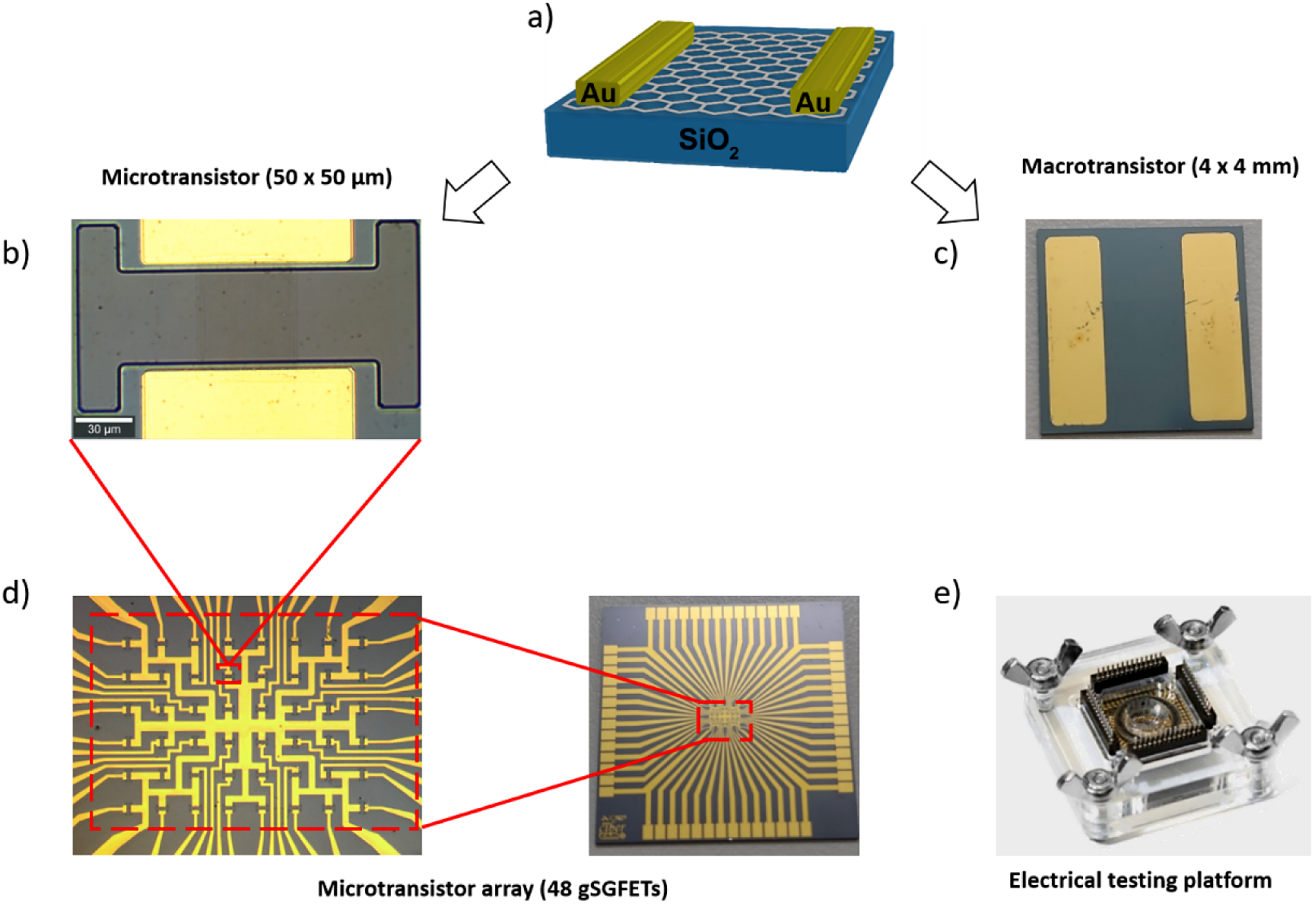
(a) Scheme of graphene transistor. (b) Microtransistor 50 × 50 μm. (c) Macrotransistor 4 × 4 mm. (d) Array of 48 microtransistors. (e) Electrical testing platform.

Two types of designs were fabricated to study gSGFET devices (Figure 1). For the experiments assessed by surface characterization physical methods, a simple structure of graphene macrotransistors was used. Macrotransistors consisted of a single 4 × 4 mm graphene active area, which is fabricated by (1) a single photolithography step with a reversal photoresist AZ5214E mask. (2) The gSGFET active areas were defined by means of an oxygen-based reactive ion etching. (3) Gold electrical contacts (200 nm) are patterned on top of transferred graphene by using a shadow mask (no resist involved).

**Figure 2:**
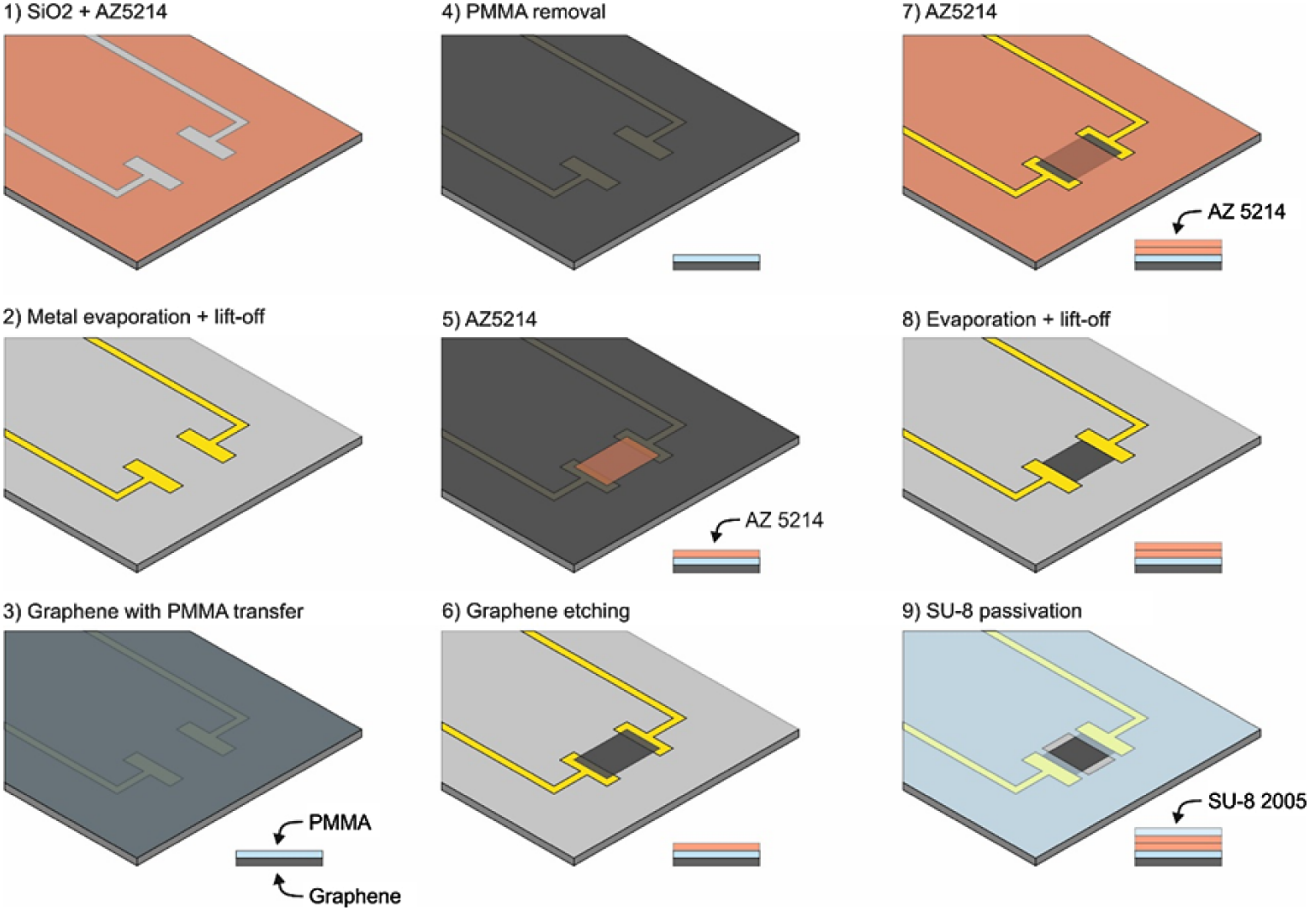
Schematic of graphene microfabrication steps with the polymer residues added. Step 3: addition of PMMA, step- 5 and 7: addition of AZ5214, step 9: addition of SU-8.

For the electrical characterization with multiple transistors, arrays of 48 graphene microtransistors with a channel area of 50 μm × 50 μm were employed. The complete processing sequence fabrication is shown in Figure 2 with the following steps: (1) a conventional lift-off process using the image reversal photoresist AZ5214E is applied to a four-inch silicon wafer covered by 2 μm-thick thermal oxide. The bottom metal layer is a thermally evaporated Ti/Au, 10/100 nm in thickness (Figure 2, steps 1, 2). (2) After graphene transfer (Figure 2, steps 3, 4), (3) the gSGFET active areas were defined by means of oxygen-based reactive ion etching based on a patterned AZ5214E resist mask (Figure 2, steps 5, 6). (4) The patterning process of the top metal layer is similar to bottom metal contact patterning, but the metal stack consisted of a Ni/Au, 20/200 nm thick film. No ultrasonication is used in this step to preserve graphene integrity (Figure 2, steps 7, 8). (5) SU-8 resist is used as the passivation layer of the gSGFETs (Figure 2, step 9). Product SU-8 2005 is an epoxy-based negative photoresist, which was patterned to passivate the metal leads while defining the graphene channel and metal contact openings.

### Surface Characterization

#### XPS

XPS characterizations of the transferred CVD graphene samples before and after solvent treatments were performed in a SPECS system (Berlin, Germany) equipped with a Phoibos 150 1D-DLD analyzer and a monochromatic Al Kα radiation source (1486.7 eV). An initial survey was carried out to analyze the corresponding elements (wide scan: step energy 1 eV, dwell time 0.1 s, pass energy 80 eV), and then, the high-resolution analysis of relevant bands was carried out with an electron exit angle of 90° (detailed scan: step energy 0.08 eV, dwell time 0.1 s, pass energy 30 eV). For all data, the Shirley-type background subtraction was used, and all curves were defined as 40% Lorentzian and 60% Gaussian. Atomic ratios were computed from experimental intensity ratios and normalized by atomic sensitivity factors. Fitting of the XPS data was done using CasaXPS 2.3.16 PR 1.6 software.

#### AFM

Surface topologies were characterized by atomic force microscopy (AFM; JPK NanoWizard II) in intermittent contact tapping mode and contact mode using a tapping etched silicon probe (TESPA-V2) with a 0.01–0.025 Ω/cm antimony (n)-doped Si, rectangular 3.8 μm thick cantilever with a nominal resonant frequency, spring constant, length, and width of 320 kHz, 42 N/m, 123 μm, and 40 μm, respectively. The obtained AFM images were analyzed in WSxM 5.0 Develop 7.0 and NanoScope Analysis 1.9

The average roughness values of the graphene surfaces were estimated by considering the root-mean-square (RMS) according to eq 1.^33^
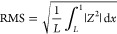
1

where *L* is the relative length of the profile, and *Z*(*x*) is the function that describes the surface profile analyzed in terms of height (*Z*) and position (*x*) of the sample over the evaluation *L*.

#### Raman Scattering: Point Spectroscopy and Mapping

Raman spectra of graphene on macrotransistors were recorded with a Renishaw Invia Raman microscope equipped with a 532 nm wavelength laser, a lens-based spectrometer with 1800 gr/mm grating, and a Peltier-cooled front-illuminated CCD (1024 px × 532 px). 4.4 Wire software was used for data analysis, processing, and representation.

For graphene microtransistors, Raman spectra were recorded with a Witec Raman spectrometer equipped with a blue laser (λ = 488 nm). The laser was focused on the sample using 50× magnification, thus providing a spatial resolution below 1 μm. The laser power was kept below 1.5 mW to avoid sample heating. A 600 lines/nm spectral grating was used, allowing a spectral resolution, pixel to pixel, of 3 cm^–1^. Raman mappings were registered over a 900 μm^2^ area using an acquisition time of 3 s. The Raman mappings were analyzed and plotted after baseline correction by means of Witec software version 5.1.

### Electrical Characterization

A measuring cell is made of PMMA and included spring loads and connectors to interface with our electronic acquisition and signal processing instrumentation (see Figure 1e). Current–voltage measurements (the transfer curves) of graphene transistors were performed in the common gate mode with a fixed drain–source voltage (*V*_ds_ = 50 mV), varying the gate–source voltage (*V*_gs_), versus Ag/AgCl reference electrode in 0.01 M PBS solution. For a better characterization of the effects of the solvents on the gSGFET performance, the charge neutrality point (CNP) and transconductance (*g*_m_) are measured.

## Results and Discussion

Polymers solvation is a complex process involving many different phenomena, which include thermodynamic and kinetic considerations, e.g., solvent–polymer penetration, polymer chain disentanglement, and the comprehension of the strength of chemical interactions between their molecules. These different aspects often counteract each other, and thus, ascertaining the best choice requires some considerations.

From a thermodynamic perspective, the Hildebrand and Hansen solubility parameters stand out as the most favored approaches for determining the optimal solvents (or combinations of solvents) for polymer solvation.^34^ These parameters are used to assess molecular interactions based on three primary attributes: polar, dispersive, and hydrogen bonding interactions. However, kinetic mechanisms also play an important role in the polymer dissolution process, as only considering favorable interactions does not guarantee an efficient dissolution. The solvent molecules need to make good physical contact with the polymer chains. Thus, their respective molecular sizes, processing temperature, and other kinetic features such as diffusion may significantly determine the actual solubility parameters.

The proper selection of solvents for residue removal depends on the polymers employed during the microfabrication process. The key steps in this process include metal evaporation, transfer of graphene, defining the active graphene area, additional metallization, and depositing the passivation layer. In this work, the commercial polymer materials used are PMMA from the graphene transfer process, phenolic polymers such as AZ resin from the photolithographic steps, and epoxy-based SU-8 from the passivation layer (Figure S1). As specific information about physicochemical properties for AZ and SU-8 polymers was not available, we used information on chemically similar polymers such as Amberol F7 and Araldit, respectively. Thus, the indicative solubility parameters of the polymers employed have the following values: δ_PMMA_ = 19.0 MPa^1/2^, δ_Amberol F-7_ = 19.0 MPa^1/2^, and δ_Araldit_ = 21.0 MPa^1/2^.^35^ It is worth noting that the closer the solubility parameters of the solute and the solvent are, the more likely is the solubility of the solute in the given solvent. In this regard, both selected solvents, EtOH and THF, exhibit solubility parameters that are acceptable for the target polymers (δ_EtOH_ = 26.0 MPa^1/2^, δ_THF_ = 18.6 MPa^1/2^).^35^ Also, they have low molecular weights, and both solvents are volatile and easy to remove. While THF is considered one of the most efficient universal solvents, EtOH can be preferred for industrial applications as it is nontoxic and more environmentally friendly. Given these considerations, we addressed an evaluation of the ability of EtOH and THF to clean polymer residues from the graphene surface in SGFETs. Initially, the impact of PMMA on gSGFET as single macrotransistors will be assessed by characterizing the graphene surface through XPS, AFM, and Raman spectroscopy. Then, the influence of the polymers PMMA, AZ, and SU-8 on the electrical properties of gSGFET as microtransistor array will be studied through their transfer curves.

### XPS Analysis

**Figure 3:**
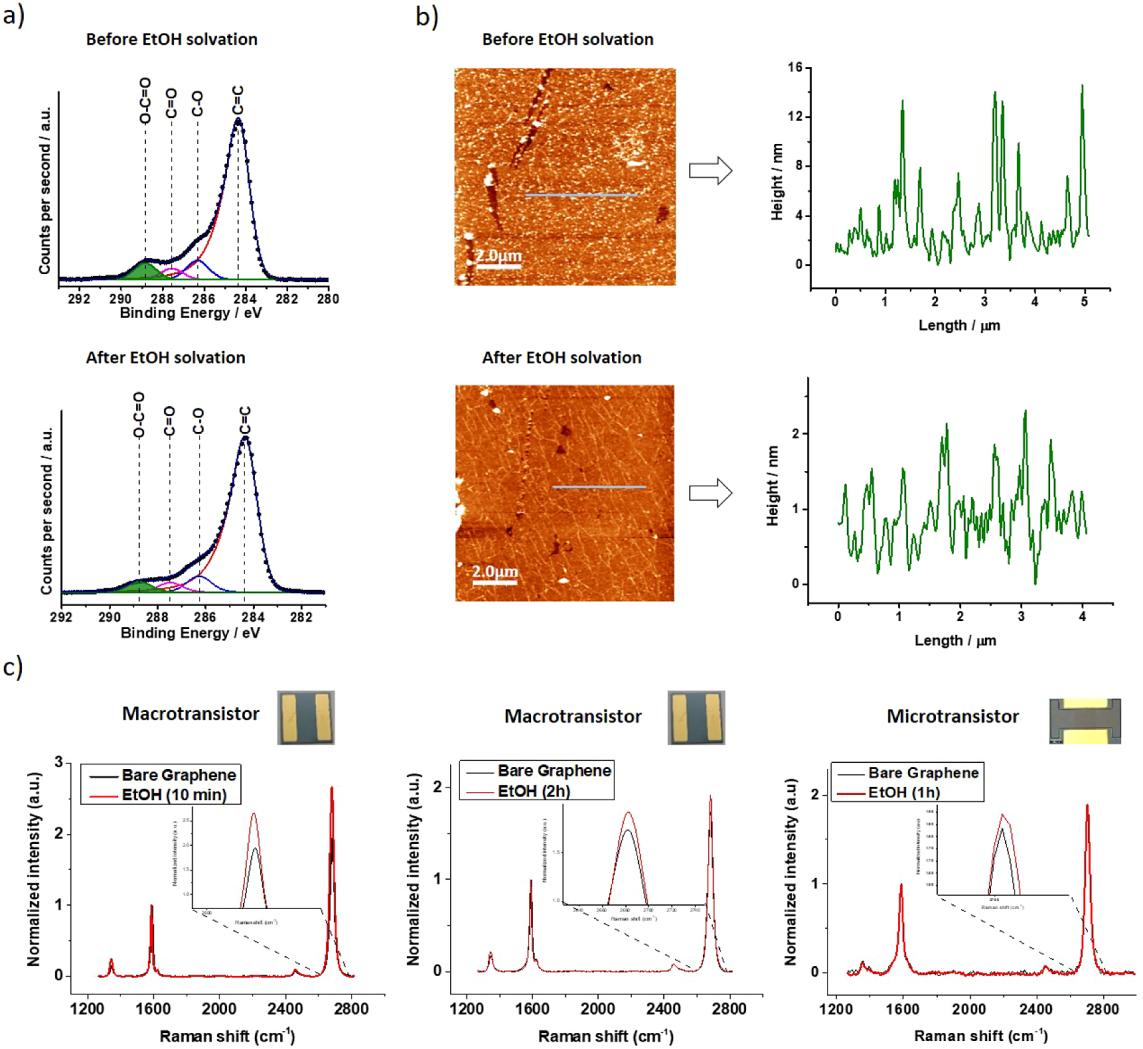
(a) Deconvoluted C 1s core levels for a graphene-based macrotransistor. Top image as-processed and bottom image after EtOH solvation for 2 h. (b) AFM images of graphene surface on a macrotransistor and height profiles before (top image) and after cleaning process (bottom image) with EtOH 2 h -(blue lines in the AFM images). (c) Averaged Raman spectra (≈1000 single-point spectra, λ_exc_ = 532 nm in 30 × 25 μm area before (black) and after (red) cleaning process of macrotransistor with EtOH 10 min (left image) and EtOH 2 h (center image). Averaged Raman spectra (≈400 single-point spectra, λ_exc_ = 532 nm) for before (black) and after 1-h EtOH treated (red) microtransistor (right image).

XPS offers evidence of polymer contamination by correlating it with the chemical bond spectrum derived from the graphene surface. The pristine graphene of microstransistors has shown a typical XPS spectra for CVD graphene.^36^ After deconvolution, the C 1s core level has shown C=C as the main component at 284.7 eV, which corresponds to the band of graphitic carbon. Oxygen-containing carbon groups such as C–O and C–O–C (≈286 eV), C=O (≈287.5 eV), and O–C=O (≈289 eV) have also appeared due to adventitious carbon and residues present on graphene (Figure 3a and Table S2). As graphene is supported on an oxidized silicon wafer, the survey spectrum also contains substrate-related contributions due to the probe depth of XPS, which is greater than the graphene film thickness (Table S1).

As mentioned above, macrotransistor fabrication implies the direct contact of graphene with PMMA during delamination and transfer processes and AZ resin for patterning purposes. It is worth noting that SU8 is not used in macrotransistors, because no passivation is used in these devices. Considering the chemical composition of the polymers used and their C 1s core levels (Figure S1), variations in the C/O atomic ratio and the increment of oxygen-containing carbon groups’ components can be used as diagnostic signals to analyze the presence of polymers on graphene before and after solvent treatments. The initially higher area of the O–C=O component compared to that of suspended monolayer graphene suggests that the contamination corresponds to PMMA residues.^36^ This inference is supported by examining the spectra of PMMA and AZ films (Figure S1). The C 1s core level of PMMA presents a prominent O–C=O component, whereas AZ does not display significant oxygen-content components as diagnostic signals. This makes AZ intrinsically more difficult to track as a source of residue at the C 1s core level. For EtOH-treated samples, a reduction of approximately 2.5% in O–C=O groups was estimated for both 10 min and 2 h of immersion (Figure 3a bottom image, Table 1, Figure S2, and Tables S1–S2). However, for THF-treated samples, it is not possible to definitively assert any eventual decrease because the initial cumulative quantity of C=O plus O–C=O was originally lower, thereby hindering the unequivocal determination of changes.

**Table 1:** Summary of Graphene Surface Characterization with XPS for the C 1s Component Before and After Solvent Treatment for 2 h with EtOH

C 1s component	% at before solvation	% at after EtOH 2 h solvation	Δ% at_(after–before)_
C=C	77.21	80.93	+3.72
C–O	9.29	8.51	–0.78
C=O	5.42	5.09	–0.33
O–C=O	8.08	5.47	–2.61

On the other hand, when quantifying C/O/Si atomic ratio before and after solvent steps, it becomes evident that an effective removal of certain amount of polymer residues has been achieved. After solvent treatment, the atomic percentage of carbon significantly decreases compared to those of silicon and oxygen in all macrotransistors. This observation was valid for both applied solvents (Figure 3a, Figures S2–S5, Table S1). Furthermore, this result leads us to conclude that the adsorption of solvent molecules is insignificant in comparison with the removal of polymer residues. Consequently, we anticipate that any potential solvent molecules adsorbed onto graphene will not exert a significant influence on the electrical outcomes.

### AFM Analysis

Observable topographic alterations occur during polymer solvation in both EtOH and THF, with notable distinctions noted in the duration of the treatment. When characterizing the nanometer-sized polymer residues in terms of roughness, a quantifiable decrease in values is noted for all solvents and durations, reaching approximately 1 nm.^37−40^

As observed in the AFM images (Figures 3b, S6–S9), its highly rough surface suggests the presence of polymer residues in the form of spots. Although these residues are present in all processed samples (both for EtOH- and THF-treated devices), their distribution and density are not entirely uniform. Particle sizes range from 2 to approximately 10 nm in height, with some clustered polymer residues of larger size also evident (Figure S10).

Upon solvation, nanometer-size defects in the form of holes or cuts are also revealed, while macroscopic detachment tends to proliferate more when THF is used as a solvent (Figure S11). This phenomenon can be attributed to a cracking process facilitated by the polymer swelling.

Ouano and Carothers have also reported similar crack initiation phenomena,^28,30^ which occur more rapidly with smaller, more efficient solvents such as methyl acetate (MA) and THF compared to the larger and less effective solvent such as methyl isobutyl ketone (MIBK). This difference is attributed to the higher diffusion rates and swelling capacity of molecules in the former solvents. It has been reported that certain polymer films crack when exposed to low-molecular-weight solvents such as MeOH, even at relatively low temperatures.^29^ These studies suggest that the “internal pressure” builds up faster than the glassy matrix can relax through gradual swelling, resulting in a catastrophic fracture outcome.

**Figure 4:**
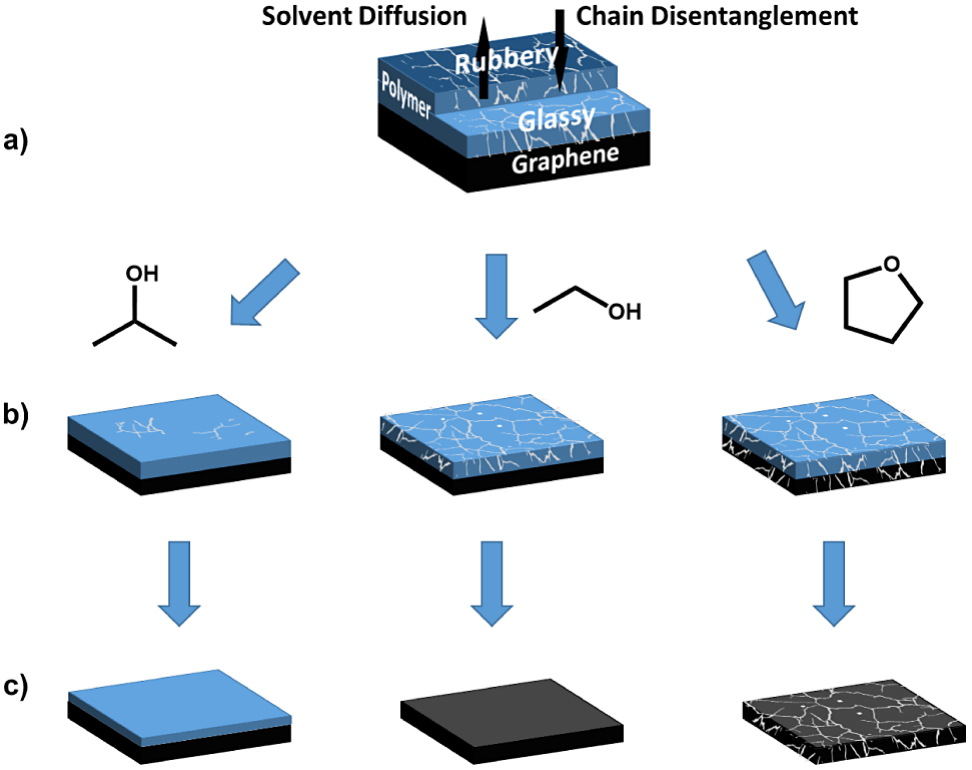
(a) Schematic of polymer residues on a graphene device. (b) Principle of one-dimensional solvent diffusion (2-propanol, EtOH, and THF from left to right) and polymer dissolution (adapted from ref. (28)). (c) Solvent-based mechanism of the solvents to penetrate and eliminate the polymer film with possible graphene cracking.

The detachment of graphene observed during the THF treatment could be attributed to mechanical damage of the polymer during its dissolution (Figure 4), which aligns with findings reported by Ouano et al.^28,30^ regarding the dissolution of PMMA in THF. The consequence of this cracking is now proven, for the first time, to affect the integrity of the graphene layer.

A summary table clearly confirms the evidence of solvation of the various polymers after both solvent treatments and the resultant morphological damage. This confirmation is evident from the results of AFM inspection and the reduction in the oxygen content in the XPS analysis (Tables 2, S1–S3).

**Table 2:** Summary of Graphene Surface Characterization upon Solvent Treatments^a^

chemical solvent	solvation time	ΔO–C=C (%) – XPS	Δroughness (%) – AFM	macroscopic damage
EtOH	10 min	–2.56	–0.37	none
EtOH	2 h	–2.61	–1.89	none
THF	10 min	–0.68	–0.80	detachment
THF	2 h	–1.02	–0.77	detachment

a Reduction in the traceable XPS peak and AFM roughness with the different solvents at different times.

### Raman Analysis

Raman spectroscopy on the different substrates was conducted to evaluate the impact of the EtOH and THF on the doping level of graphene and its crystal lattice, first on macrotransistors and second on microtransistors (Figures 3c, S12–S13). For macrotransistors, the Raman spectra of produced bare graphene on SiO_2_ substrate showed a D peak at ∼1.350 cm^–1^, narrow 2D peak at ∼2.680 cm^–1^ (fwhm = 37 cm^–1^ by fitting a single Lorentzian peak), and an average 2D-to-G intensity ratio (*I*_2D_/*I*_G_) of 1.84. This data is consistent with mainly monolayer graphene.^41^

After cleaning with EtOH and THF, some of the physical characteristics of graphene are affected by solvent treatment. It is important to note that doping relates to the concentration of electrons or holes within graphene, which can arise from charged and polar impurities, such as polymer residues. These variations in the electron or hole density lead to electronic modifications in graphene. Raman spectroscopy demonstrates sensitivity to these electronic changes and can effectively track doping in graphene. In particular, valuable information can be obtained from the ratio of the 2D and G bands’ intensities (*I*_2D_/*I*_G_) as it exhibits dependence on doping levels.^42,43^ A decrease in p-doping leads to a significant increase in *I*_2D_/*I*_G_ (Table S4).^42^ Notably, in the case of macrotransistors treated with EtOH, there is an observed increase in *I*_2D_/*I*_G_, with values of 0.71 and 0.19 for treatments lasting 10 min and 2 h, respectively (Figure 3c, left and center images). Moreover, a microtransistor device, after 1 h of EtOH treatment, showed an increase in normalized *I*_2D_/*I*_G_ of approximately 0.06 arbitrary units (Figure 3c, right image). This experimental evidence demonstrates the reduction of polymeric residues on the graphene surface by using EtOH as a solvent in terms of hours. It is worth noting that not relevant variations in *I*_D_/*I*_G_ ratio for graphene after treatment with EtOH and THF (∼0.13) confirms the preservation of its crystalline structure.

### Electrical Analysis

The residual polymer contamination is detrimental to graphene electronic devices, e.g., when the extreme graphene characteristics are pursued for fundamental studies.^8^ In those cases, usually, backgated devices are preferable to evaluate the electrical parameters, mainly the electronic mobility using hall-bars.^21^ However, in sensing and biosensing applications, usually performed by solution gated devices, some other electrical parameters are relevant, such as the transfer characteristics, which provide information on the doping level through the CNP or the carrier mobility by means of the transconductance. In addition, it is crucial to have a certain homogeneity in these electric metrics parameters, either for before–after analysis or real-time measurements in which preliminary calibration is required.^44^ In this regard, the presence of polymer contamination on graphene layer has a tremendous impact on the gSGFET electrical performance.^9,10^ As previously detailed, the use of solution gated devices adds an extra polymeric layer in order to isolate the contacts from the graphene-sensing channel that is in contact with the electrolyte solution. This additional passivation layer is unnecessary in back-gated devices, leading to limited literature addressing its electrical impact concerning contamination.

Thus, the impact of the passivation layer in the electrical performance of solution gated graphene microtransistors before and after the polymer residue solvation procedure has been evaluated. The electrical characterization was mainly performed by evaluating the *I*–*V* transfer curve, measuring the drain-source current (*I*_ds_) versus the gate-source voltage (*V*_gs_), whose minimum gives access to the CNP. This parameter is mainly affected by the intrinsic doping level of graphene and the surface charges on the graphene/solution interface. Therefore, the adsorption or desorption of charged species and the chemical alterations such as ion concentrations,^45^ pH variations,^46^ or chemical functionalization^47,48^ can be precisely evaluated by these CNP variations.^47^

**Figure 5:**
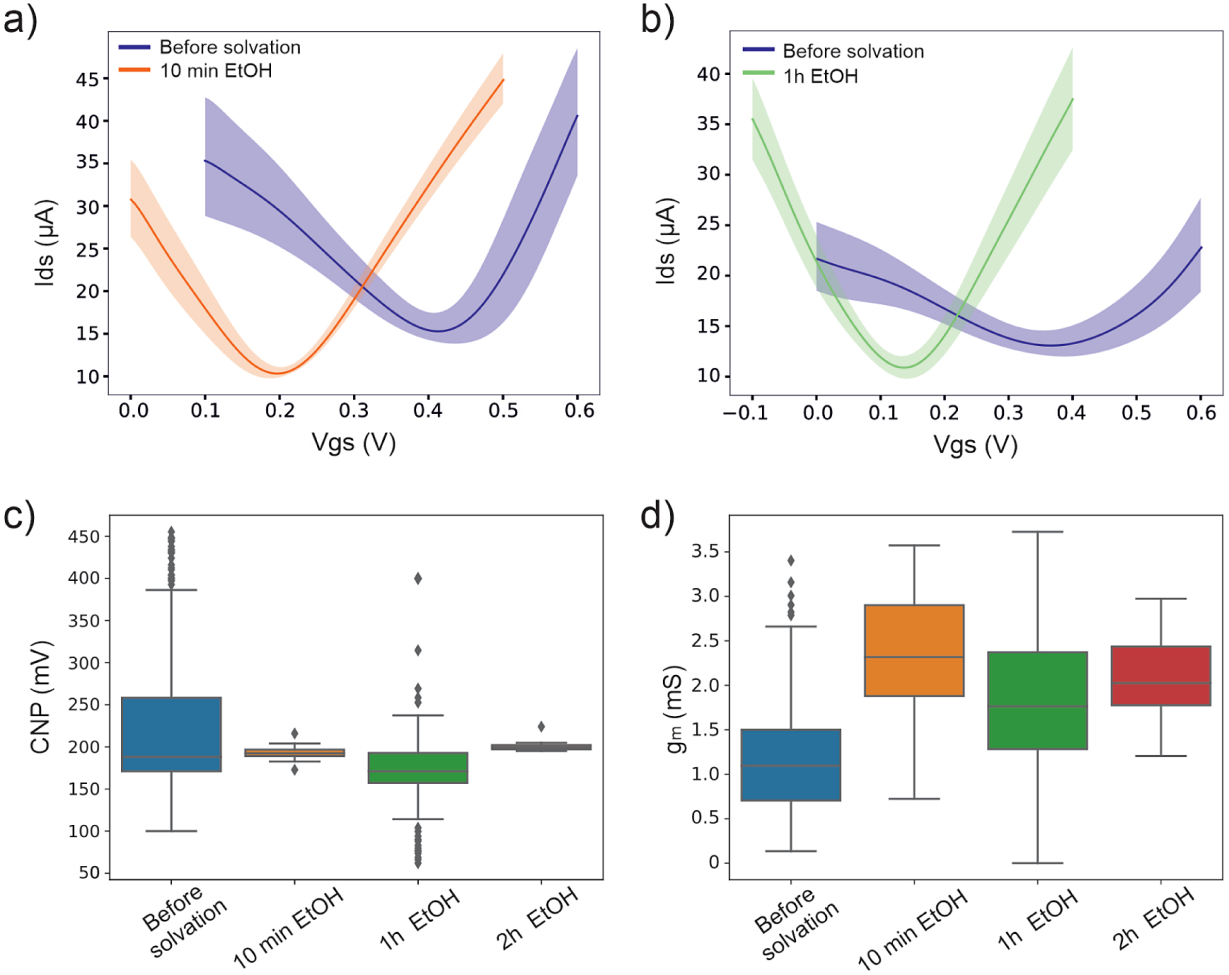
(a) Current to voltage curve of one chip with 48 transistors before and after ethanol treatment for 10 min. (b) Current to voltage curve of one chip with 48 transistors before and after ethanol treatment for 1 h. (c) Boxplot of CNP of different chips with all of the treatments carried out. (d) Boxplot of the transconductance (*g*_m_) of different chips with all the treatments carried out.

The impact of polymer solvation on the entire transfer characteristics is evident in Figure 5a,b, where EtOH treatments of 10 min and 1 h were applied to two distinct microtransistor arrays, respectively. In the first case, the CNP values were approximately 0.4 V, and postsolvation, the CNP values shifted toward 0.2 V. Similarly, with the 1-h ethanol treatment, the CNP shifted from 0.35 to 0.12 V, reducing the CNP value by approximately 0.2 V in both cases. As mentioned, the layer of polymer residues is not always uniform, resulting in varying electrical behavior among transistors where the CNP value exhibits variability prior to solvent treatment.

Figure 5c evaluates the alterations in the CNP values before and after polymer solvation with ethanol treatment at different times. The utilization of different 48-gSGFET microtransistor arrays allows simultaneous measurements, facilitating multiple-replicas, which is essential to acquire statistically robust data.^49^ The results depicted in Figure 5c confirm that EtOH solvation of polymer residues after the entire fabrication process significantly reduces the variability in the CNP values. A 1-h EtOH treatment has demonstrated to be the optimal duration for effectively covering and ensuring the maximum reduction of various residue amounts. This reduction is crucial for enhancing the electrical performance of the devices, particularly for biosensing calibration.

Similarly, the same evaluation was performed with THF as the solvation agent (Figure S14). However, after the treatment, we observed some effects on the passivation layer that isolates the contacts. This impact becomes apparent after repeated measurements and manifests as the penetration of the electrolyte solution through the contacts, rendering the transistors unusable for extended periods (Figure S15). In addition, as discussed earlier, the use of THF is limited for some applications if flexible substrates are needed to fabricate the whole device because the use of this solvent can degrade the adhesion between layers, provoking the electrolyte solution penetration through the contacts.

Other relevant indicators of the affectation of the polymer in the electrical performance are the transconductance and the shape of the *I*–*V* curve. Additionally, in the transconductance analysis, a notable increase of 2.5-fold in Figure 5d is observed. The transconductance, which is the slope of the linear part of the transfer curves, can also be affected due to its dependency on the mobility of the charge carriers and the impurities that alter the structural quality of the graphene surface. When a polymer layer remains on the surface of the graphene, it impedes the proper modulation of the current through the graphene channel. This is translated into a low transconductance (low *I*/*V* curve slope), mainly observed in Figure 5b. High transconductance is essential for achieving a large sensitivity in chemical and biological sensors since it reflects small modulations as an analog change in their current response. Thus, in addition to CNP variability, the presence of polymers on the graphene channel can affect the transconductance. In particular, an asymmetry in the transconductance curve indicates different mobilities and scattering cross sections for electrons and holes,^50^ which has been precisely correlated, for instance, to SU-8 passivation.^51,52^ Additionally, it must be considered that the amount of polymer residues can vary from wafer to wafer but also among samples of the same wafer, having a higher impact toward the modulation of the channel than others (Figure 5b). Thus, this notable increase (Figure 5d) indicates promising prospects for higher sensitivity, as discussed earlier.

## Conclusions

In this work, we investigate the efficacy of EtOH and THF as solvents for reducing polymer residues from graphene surfaces, a critical step for ensuring the optimal performance of solution-gated field-effect transistors. We emphasize the importance of selecting solvents that closely match the solubility parameters of the residues while preventing any damage such as graphene cracking or detachment and ensuring compatibility with all device materials.

Both solvents demonstrate significant effectiveness in reducing residues following microfabrication processes, such as photolithography. However, despite the effectiveness of THF, its utilization seems to compromise the structural integrity of graphene and the polymer passivation layer in gSGFET devices. In contrast, EtOH offers practical advantages, including compatibility with various device layers and scalability at the wafer level, rendering it as a preferred and environmentally friendly solvent option.

In addition, EtOH’s solvation capabilities have been demonstrated to significantly decrease the dispersion of CNP and transconductance, with an optimized solvation time of 1 h effectively reducing the doping level caused by polymer residues. This efficiency in residue removal not only enhances the electrical performance but also offers precision and reliability in graphene-based technologies, particularly in sensing and biosensing applications.

It is important to highlight that the use of ethanol not only enhances device performance but also contributes to a more sustainable and socially responsible approach to semiconductor processing, being aligned with principles of green chemistry. The integration of efficient and environmentally friendly solvent options will play a pivotal role in advancing graphene-based technologies while minimizing their environmental footprints and ensuring human safety.

## Supporting Information Available

A set of XPS spectra and AFM images comparing graphene devices before and after solvent treatment; histograms extracted from AFM images; Raman spectra before and after solvent treatment; summary tables of XPS and AFM analysis with different solvent treatments; electrical evaluations of devices treated with THF; device images after THF treatment; and a comparative table of graphene cleaning methodologies (PDF)
