# Supplementary material for: Ethanol Solvation of Polymer Residues in Graphene Solution-Gated Field Effect Transistors
Source: ACS Sustain Chem Eng. 2024 Jun 4;12(24):9133–43. doi: 10.1021/acssuschemeng.4c01538 (PMC11191359; doi:10.1021/acssuschemeng.4c01538)
Supplement: Supplementary file 1 — sc4c01538_si_001.pdf [file sc4c01538_si_001.pdf]

Supporting Information

Ethanol solvation of polymer contamination in graphene solution-gated field effect transistors

Juan Pedro Merino[1,†], Sergi Brosel-Oliu[2,†], Gemma Rius[2], Xavi Illa[3,2], Manuel Vázquez Sulleiro[4,5], Elena del Corro[6], Eduard Masvidal-Codina[6,2,3], Andrea Bonnaccini Calia[6], Jose Antonio Garrido[6,7], Rosa Villa[2,3], Anton Guimerà-Brunet[2,3], Maurizio Prato[1,4,8,*], Alejandro Criado[5,*], Elisabet Prats-Alfonso[3,2,*]

[†] These authors contributed equally to this work

*Correspondence: mprato@cicbiomagune.es (M.P.); a.criado@udc.es (A.C.); elisabet.prats@csic.es (E. P-A.)

Affiliations

1 Center for Cooperative Research in Biomaterials (CIC biomaGUNE), Basque Research and Technology Alliance (BRTA), Paseo de Miramon 194, 20014 Donostia-San Sebastián, Spain.
2 Institute of Microelectronics of Barcelona (IMB-CNM, CSIC), Campus UAB, 08193, Bellaterra, Spain
3 Centro de Investigación Biomédica en Red de Bioingeniería, Biomateriales y Nanomedicina, Instituto de Salud Carlos III
4 Department of Chemical and Pharmaceutical Sciences, University of Trieste, Via L. Giorgieri 1, 3412 7 Trieste, Italy
5 Universidade da Coruña, CICA – Centro Interdisciplinar de Química e Bioloxía, Rúa as Carballeiras, 15071 A Coruña, Spain.
6 Catalan Institute of Nanoscience and Nanotechnology (ICN2), CSIC and BIST, Campus UAB, Bellaterra, Barcelona, Spain
7 ICREA Pg. Lluís Companys 23, Barcelona 08010, Spain
8 Ikerbasque, Basque Foundation for Science, 48013 Bilbao, Spain

Table of contents

1. XPS analysis S2
2. AFM analysis S7
3. Raman analysis S10
4. Summary Tables S11
5. Electrical Characterization S12
6. Comparative Table S13
7. References S14

1. XPS análisis

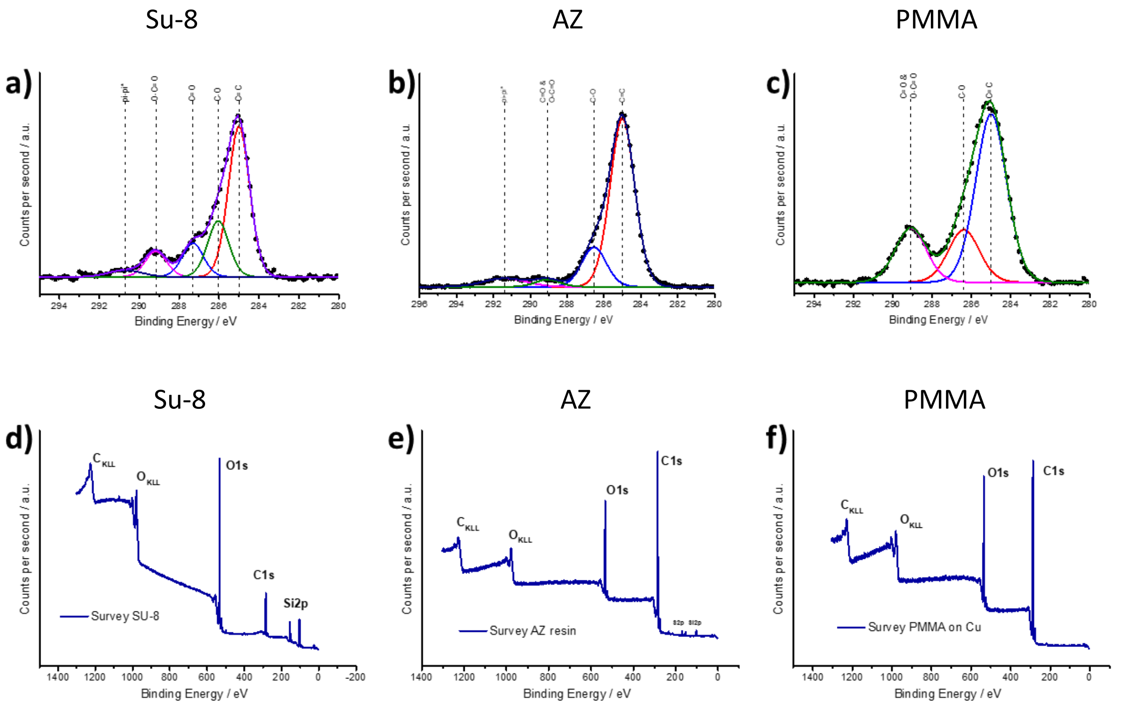

Figure S1. Deconvoluted C1s core levels and survey spectra respectively for epoxy resin SU-8 (a and d), epoxy resin AZ (b and e) and PMMA (c and f).

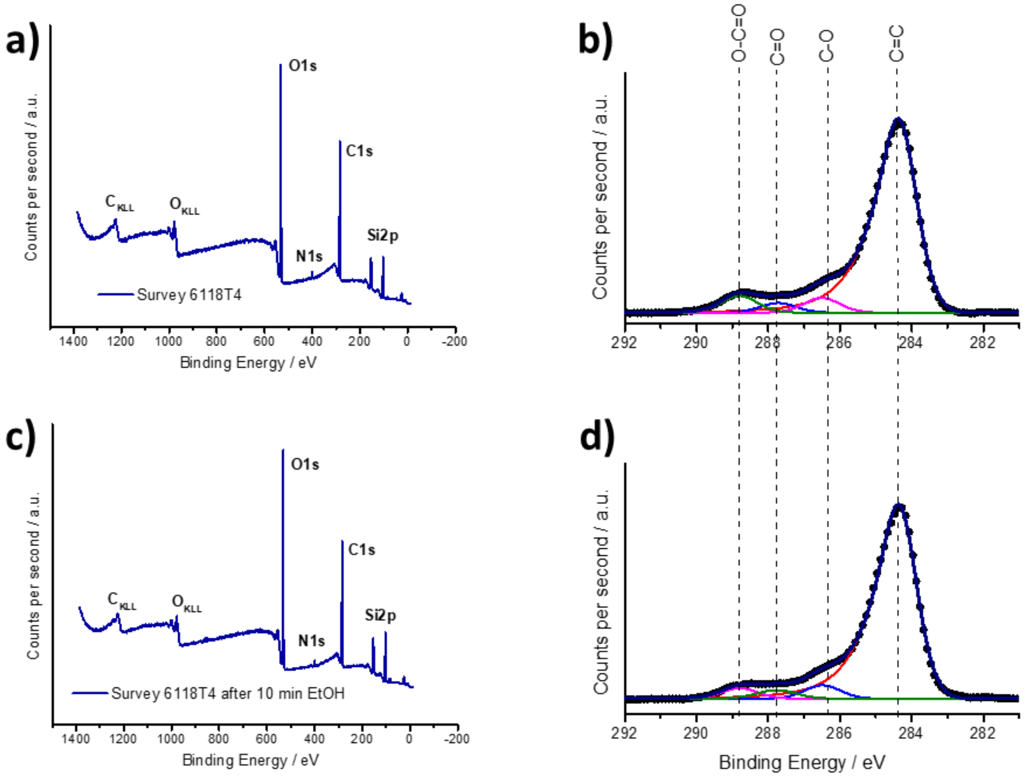

Figure S2. Survey spectra and deconvoluted C1s Core levels respectively of macrotransistor a, b) before and c, d) after 10 min EtOH treatment.

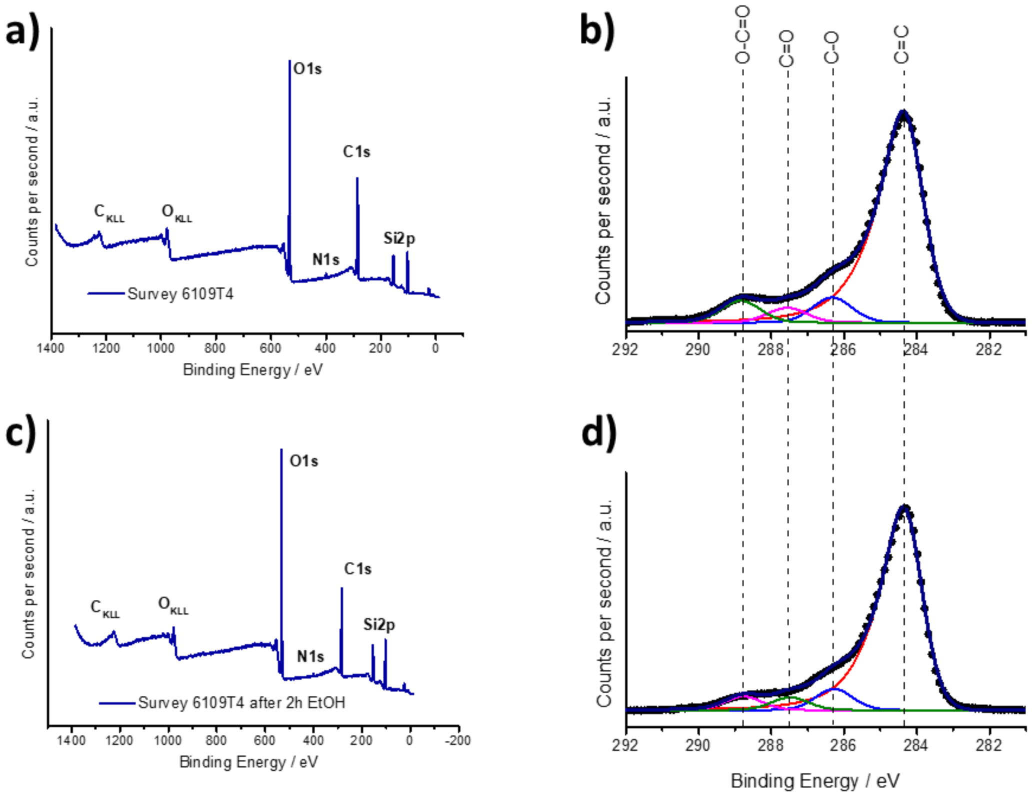

Figure S3. Survey spectra and deconvoluted C1s Core levels respectively of macrotransistor a, b) before and c, d) after 2 h EtOH treatment.

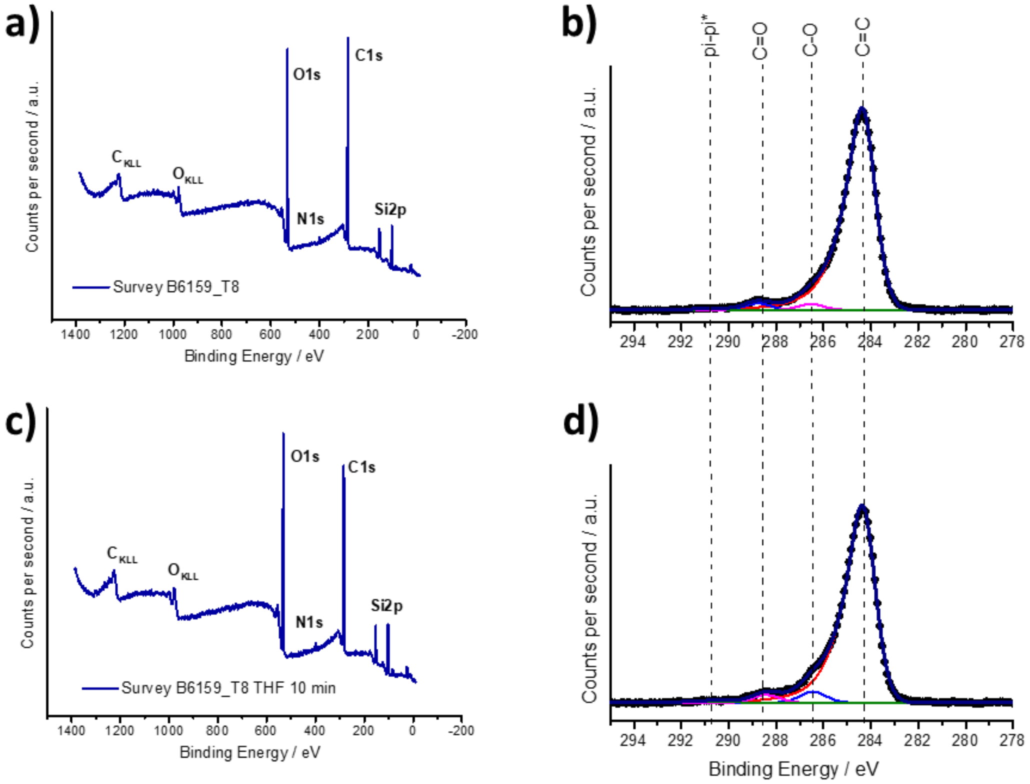

Figure S4. Survey spectra and deconvoluted C1s Core levels respectively of macrotransistor a, b) before and c, d) after 10 min THF treatment.

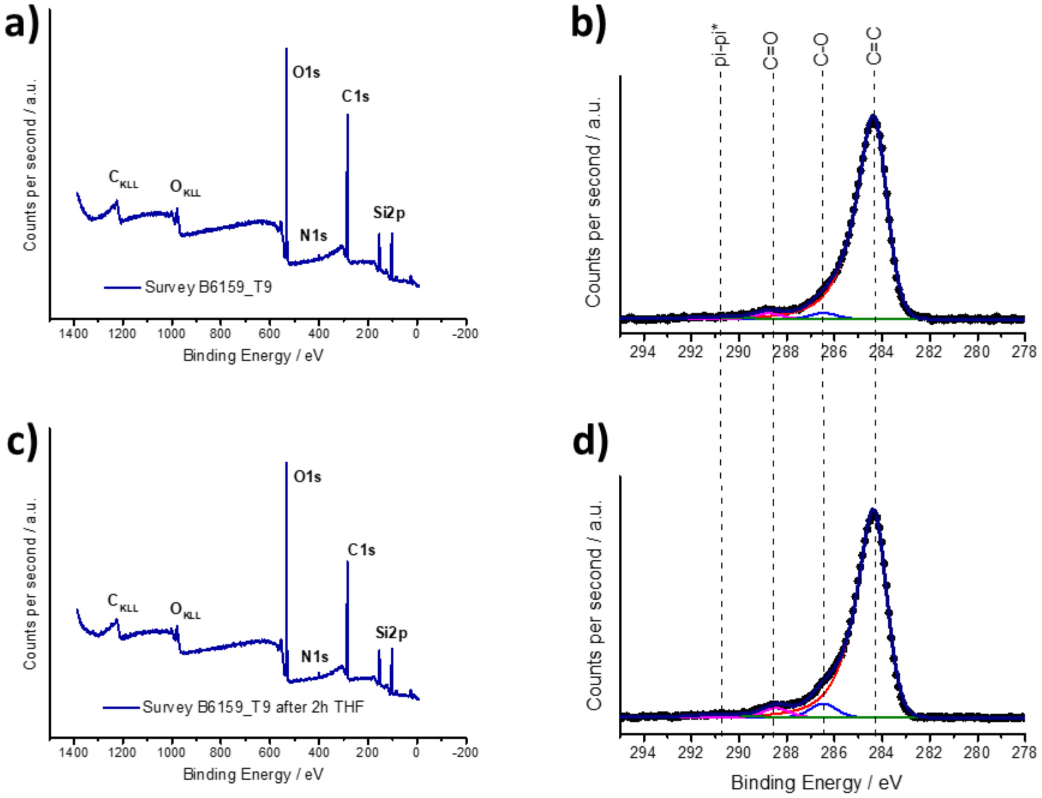

Figure S5. Survey spectra and deconvoluted C1s Core levels respectively of macrotransistor a, b) before and c, d) after 2h THF treatment.

2. AFM analysis

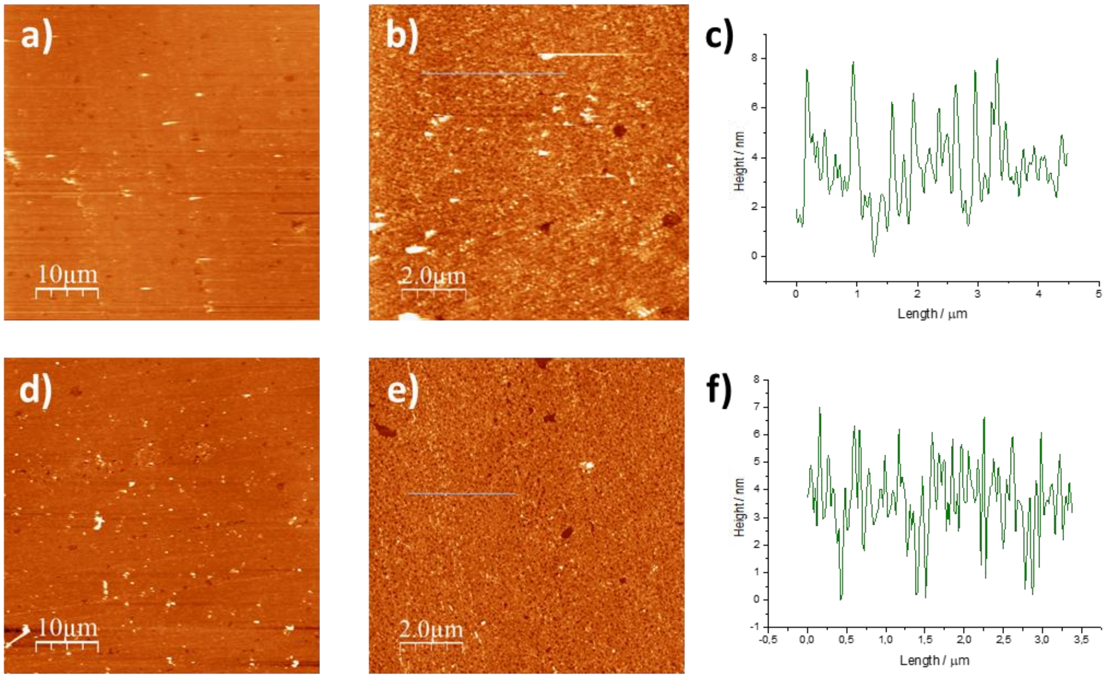

Figure S6. AFM images of graphene surface on a macrotransistor a, b) before and d, e) after cleaning process with EtOH 10 min at different magnifications. AFM height profiles c) before and f) after cleaning process with EtOH 10 min (blue lines in b and e images respectively).

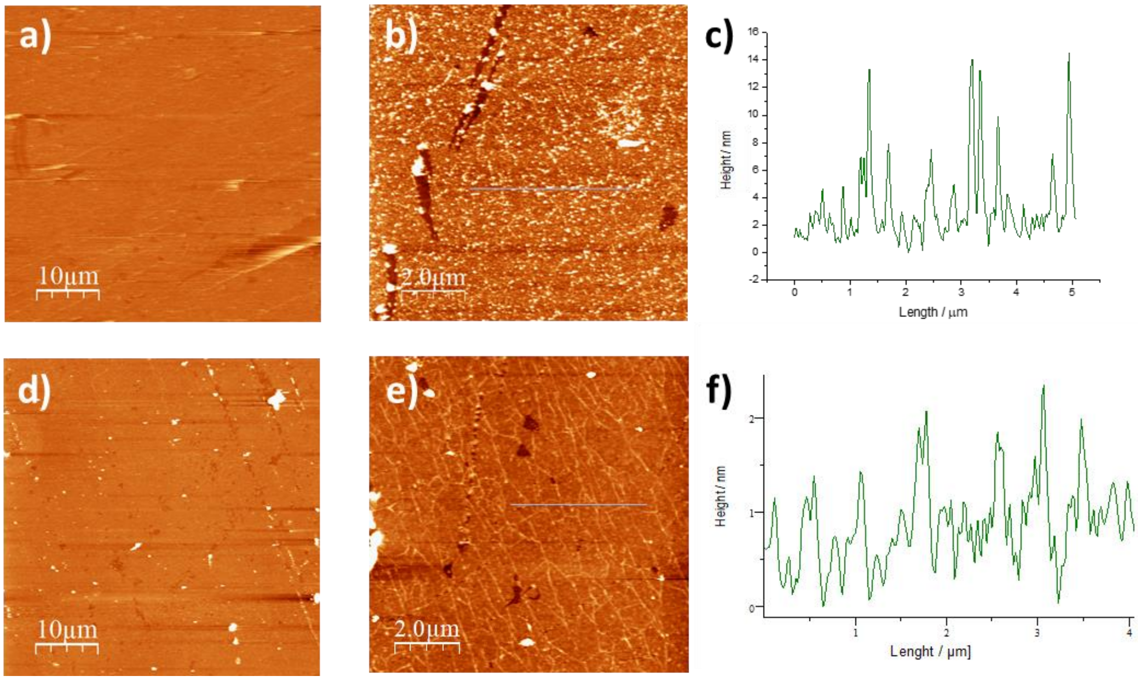

Figure S7. AFM images of graphene surface on a macrotransistor a, b) before and d, e) after cleaning process with EtOH 2h min at different magnifications. AFM height profiles c) before and f) after cleaning process with EtOH 2h min (blue lines in b and e images respectively).

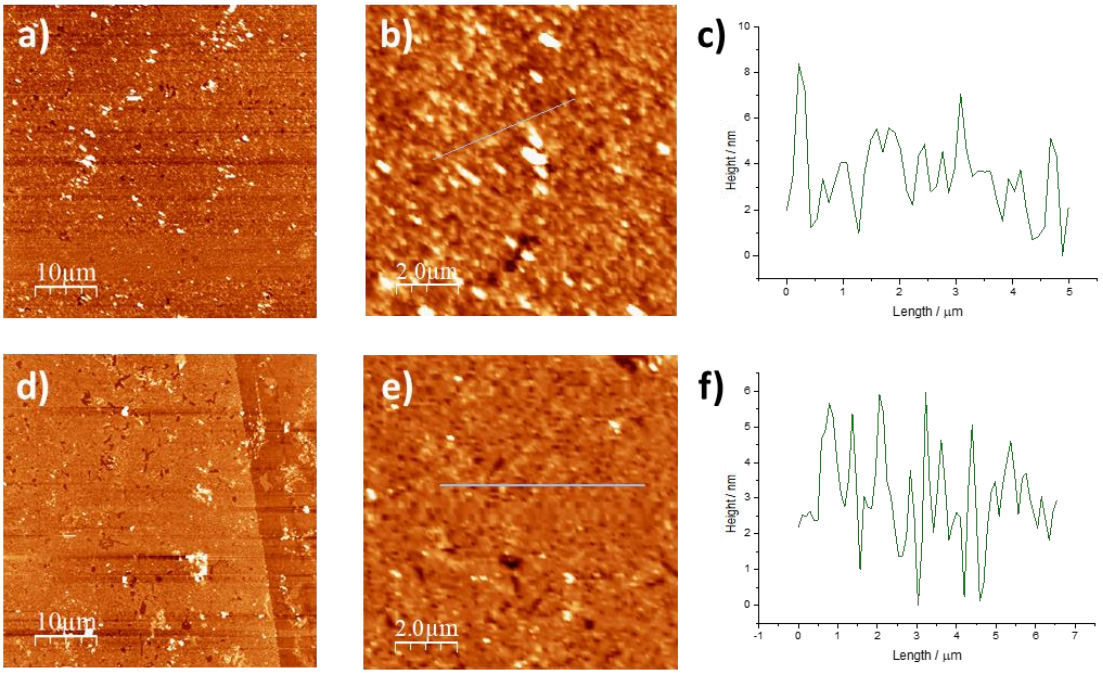

Figure S8. AFM images of graphene surface on a macrotransistor a, b) before and d, e) after cleaning process with THF 10 min at different magnifications. AFM height profiles c) before and f) after cleaning process with THF 10 min (blue lines in b and e images respectively).

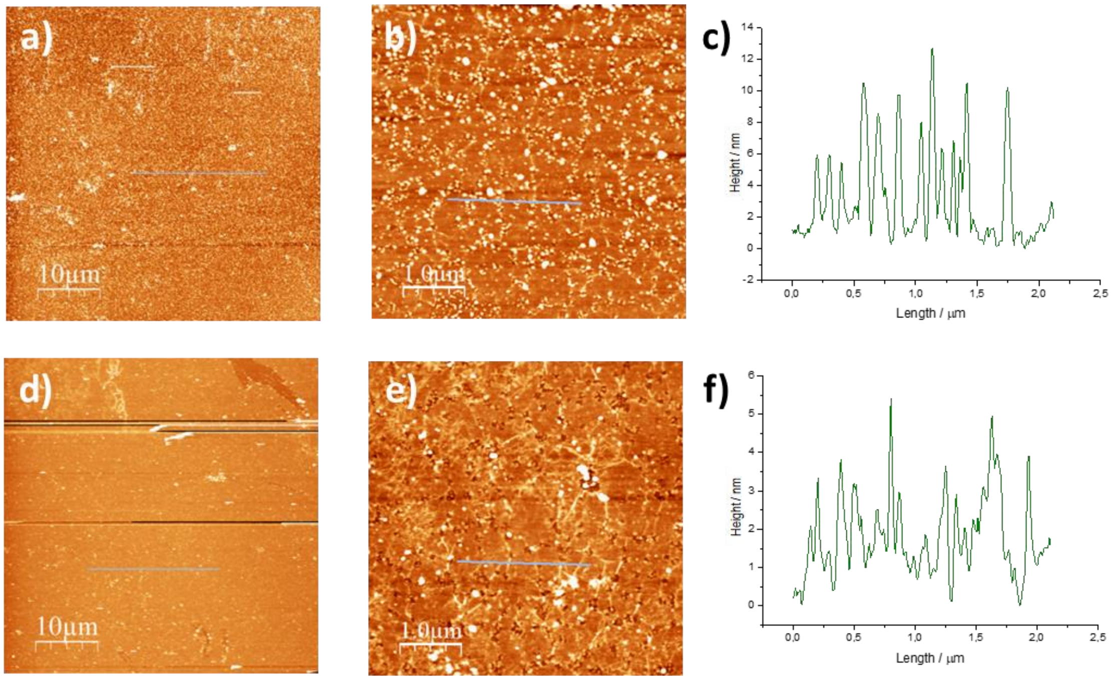

Figure S9. AFM images of graphene surface on a macrotransistor a, b) before and d, e) after cleaning process with THF 2h min at different magnifications. AFM height profiles c) before and f) after cleaning process with THF 2h min (blue lines in b and e images respectively).

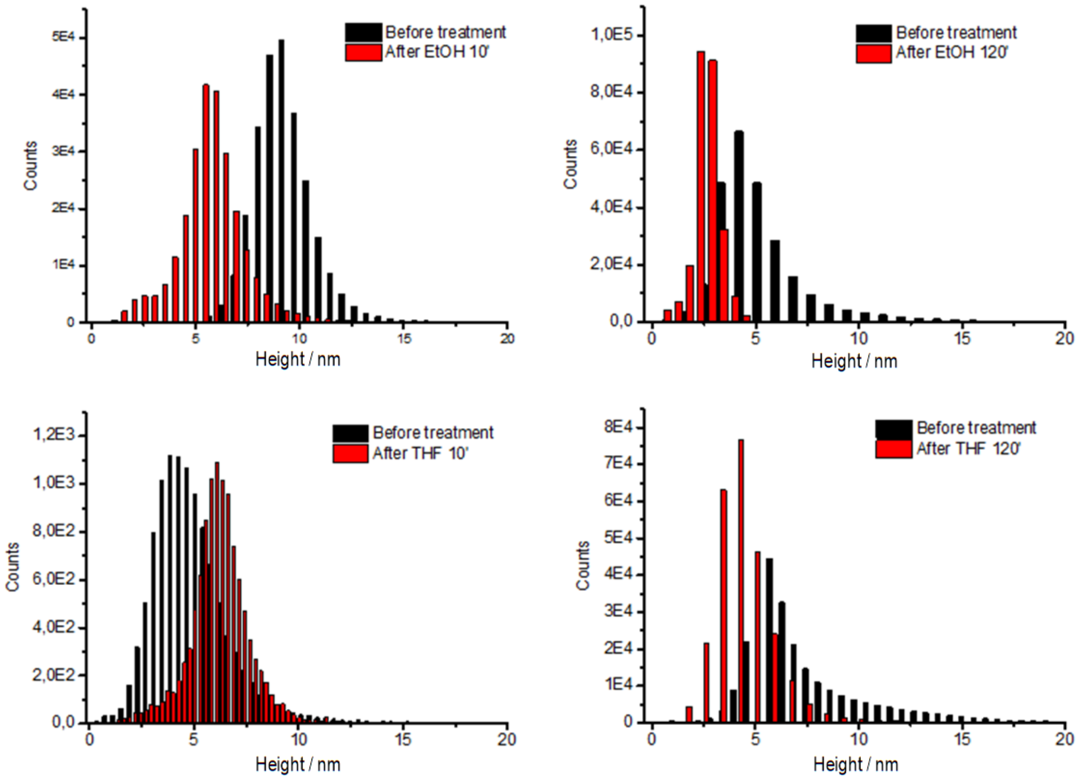

Figure S10. Histogram of heights obtained from the figures S7-S10 50 µm^2 AFM images for macrotransistors before and after treatment with EtOH and THF.

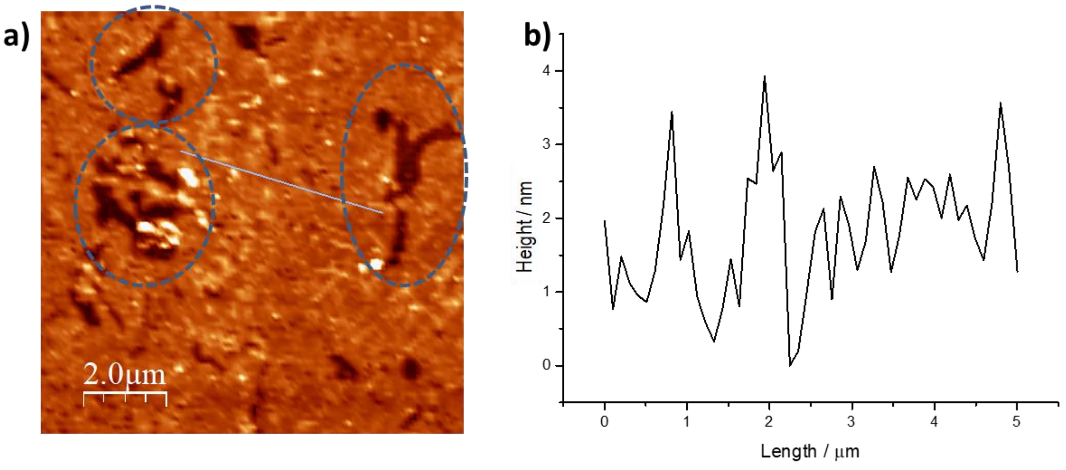

Figure S11. AFM image of graphene surface on a macrotransistor a) detachment detail and b) profile after 10 min THF treatment.

3. Raman analysis

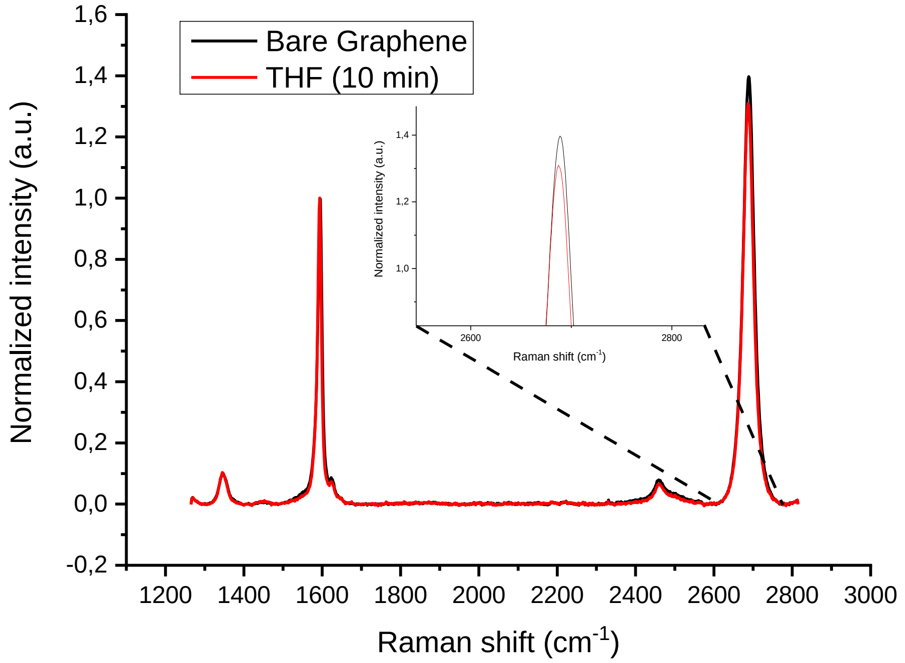

Figure S12. Averaged Raman spectra (≈1000 single-point spectra, λ_{exc} = 532nm in 30x25 µm^2 area before and after cleaning process of macrotransistor with THF 10 min.

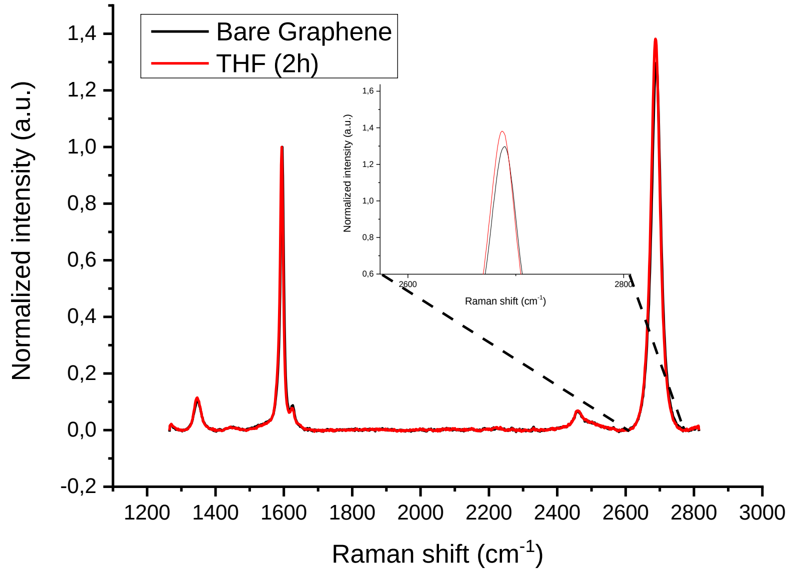

Figure S13. Averaged Raman spectra (≈1000 single-point spectra, λ_{exc} = 532nm in 30x25 µm^2 area before and after cleaning process of macrotransistor with THF 2h.

4. Summary tables

Table S1. Atomic percentage obtained by XPS analysis for the different macrotransistors before and after cleaning by different treatment conditions.

Treatment (Solvent/time)	C / at%			O / at%			Si / at%		
	Before	*After*	*Δ*	*Before*	*After*	*Δ*	*Before*	*After*	*Δ*
EtOH/10 min	54.85	50.59	4.36	27.61	28.18	-0.57	17.54	21.24	-3.7
EtOH/2 h	49.13	42.83	6.30	30.87	32.44	-1.57	20.00	24.73	-4.73
THF/10 min	64.34	60.85	3.49	19.84	22.23	-2.39	15.83	16.92	-1.09
THF/2 h	55.89	53.00	2.89	24.80	26.34	-1.54	26.34	20.66	-1.35

Table S2. C1s components for the different macrotransistors before and after cleaning by different treatment conditions.

Treatment (Solvent/time)	C1s component	Position / eV		At. ratio / %		
		Before	*After*	*Before*	*After*	*Δ*
EtOH/10 min	C=C	284.37	284.37	82.23	85.08	-2.85
	C-O	286.51	286.49	6.36	5.99	0.37
	C=O	287.73	287.78	4.20	4.27	-0.07
	O-C=O	288.81	288.79	7.22	4.66	2.56
	pi-pi*	-	-	-	-	-
EtOH/2 h	C=C	284.37	284.37	77.21	80.93	-3.72
	C-O	286.33	286.28	9.29	8.51	0.78
	C=O	287.57	287.48	5.42	5.09	0.33
	O-C=O	288.85	288.77	8.08	5.47	2.61
	pi-pi*	-	-	-	-	-
THF/10 min	C=C	284.37	284.37	93.64	90.13	3.51
	C-O	286.53	286.45	2.75	5.18	-2.43
	C=O	288.77	288.41	3.14	3.63	-0.49
	O-C=O	-	-	-	-	-
	pi-pi*	290.78	290.78	0.47	1.05	-0.58
THF/2 h	C=C	284.37	284.37	92.88	87.80	5.08
	C-O	286.47	286.47	2.81	5.86	-3.05
	C=O	288.78	288.45	2.67	4.01	-1.34
	O-C=O	-	-	-	-	-
	pi-pi*	290.78	290.78	1.64	2.33	-0.69

Table S3. AFM roughness for the different macrotransistors before and after cleaning by different treatment conditions.

Treatment (Solvent/time)	AFM roughness /nm		
	Before	*After*	*Δ (before-after)*
EtOH/10 min	2.03	1.66	0.37
EtOH/2h	3.07	1.18	1.89
THF/10 min	-	-	-
THF/2h	2.38	1.17	1.21

Table S4. I_{2D}/I_G of graphene in macrotransistors before and after cleaning by different treatment conditions.

Treatment (Solvent/time)	Transistor dimension	I_{2D}/I_G		
		Before	*After*	*Δ (after-before)*
THF/10 min	Macrotransistor	1.40	1.31	-0.09
THF/2h	Macrotransistor	1.30	1.38	0.08
EtOH/10 min	Macrotransistor	1.95	2.66	0.71
EtOH/2h	Macrotransistor	1.73	1.92	0.19
EtOH/1h	Microtransistor	1.83	1.86	0.06

5. Electrical evaluation of microtransistors before and after THF treatment.

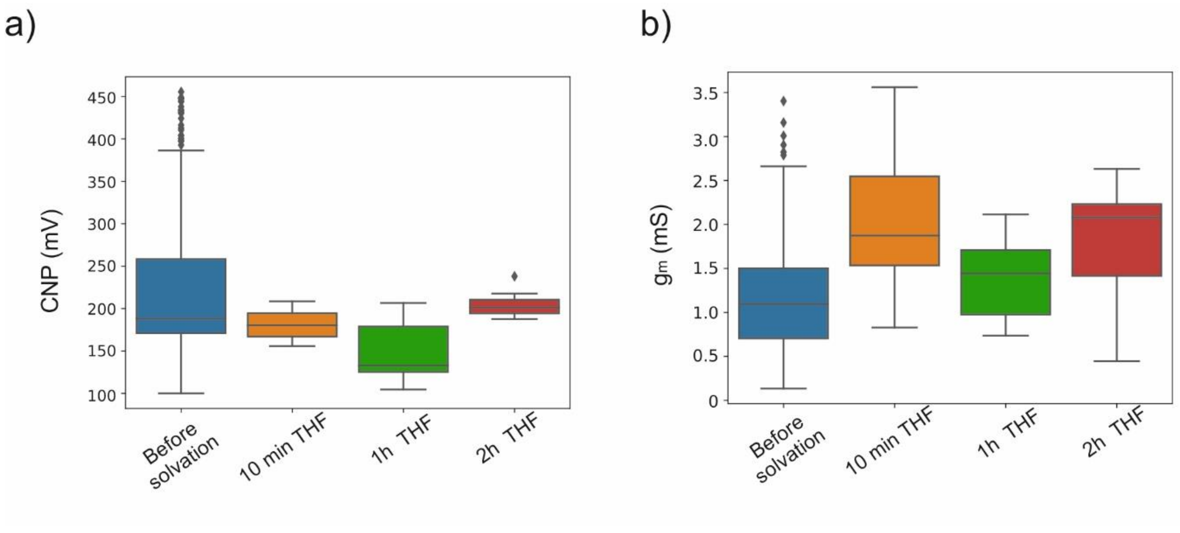

Figure S14. Boxplots of Gm (transconductance) from the different microtransistors.

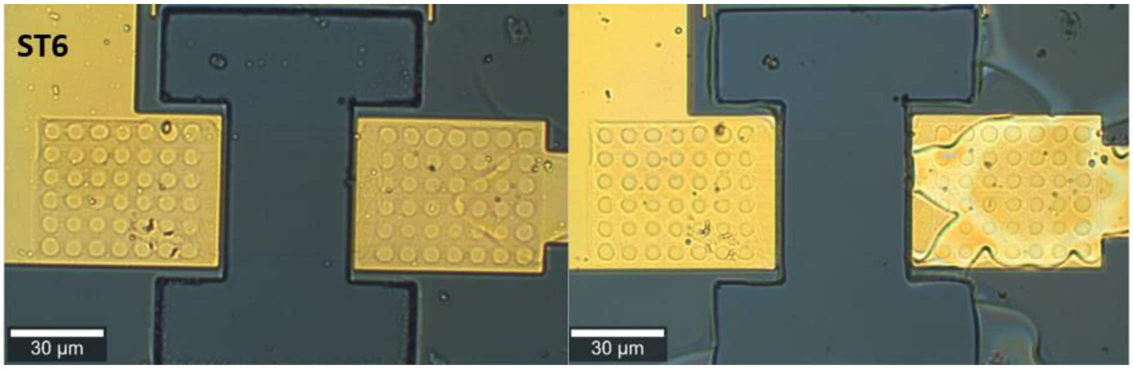

Figure S15. Transistors contacts image: left before THF treatment, right after THF treatment with detachment of the polymer passivation on the contact area. (Different transistor design from the described in the experimental section)

6. Comparative Table of graphene cleaning methodologies.

Table S5. I_{2D}/I_G of graphene in macrotransistors before and after cleaning by different treatment conditions.

Type	Advantage	Disadvantage	REF
Mechanical Cleaning (tip AFM)	Very effective	Costly, non-scalable (only small areas)	[1]
Current Induce desorption	Effective	Non-scalable (one device at a time)	[2]
Exposure to ozone	Scalable	Damage graphene	[3]
Exposure to plasma	Scalable	Damage graphene	[4]
High temperature annealing	Effective	Very Aggressive to graphene lattice	[5]
Electrostatic force	Effective	Very Aggressive to graphene lattice	[6]
Solvents: Dimethylacetamide	Simple, scalable, cost-efficient	Harsh solvents: toxic to health and environment	[7]
Solvents: Dioxolane	Simple, scalable, cost-efficient	Harsh solvents: toxic to health and environment	[8]
Solvents: 2-propanol	Simple, scalable, cost-efficient	Not fully polymer removal	[9]
Solvents: Acetone	Simple, scalable, cost-efficient	Leave organic residues	[9]
Solvents: NMP	Simple, scalable, cost-efficient, very effective	Not fully compatible with passivation layers, adsorption on graphene lattice	[10]
Solvents: Methanol	Simple, scalable, cost-efficient	Polymer cracking possible affectation Graphene lattice	[11,12]
Solvent THF	Simple, scalable, cost-efficient, very effective	Affectation integrity graphene lattice and passivation layer	This work
Solvent Ethanol	Simple, scalable, cost-efficient, very effective, environmentally friendly, safe, green solvent	Needs further studies with other polymers	This work

References

(1) Goossens, A. M.; Calado, V. E.; Barreiro, A.; Watanabe, K.; Taniguchi, T.; Vandersypen, L. M. K. Mechanical Cleaning of Graphene. *Appl. Phys. Lett.* **2012**, *100* (7), 73110. https://doi.org/10.1063/1.3685504.

(2) Moser, J.; Barreiro, A.; Bachtold, A. Current-Induced Cleaning of Graphene. *Appl. Phys. Lett.* **2007**, *91* (16), 163513. https://doi.org/10.1063/1.2789673.

(3) Prudkovskiy, V. S.; Katin, K. P.; Maslov, M. M.; Puech, P.; Yakimova, R.; Deligeorgis, G. Efficient Cleaning of Graphene from Residual Lithographic Polymers by Ozone Treatment. *Carbon N. Y.* **2016**, *109*, 221–226. https://doi.org/10.1016/j.carbon.2016.08.013.

(4) Arias-Zapata, J.; Ferrah, D.; Mehedi, H.; Cunge, G.; Zelsmann, M. Effective Patterning and Cleaning of Graphene by Plasma Etching and Block Copolymer Lithography for Nanoribbon Fabrication. *J. Vac. Sci. Technol. A* **2018**, *36* (5), 05G505. https://doi.org/10.1116/1.5035333.

(5) Xie, W.; Weng, L.-T.; Ng, K. M.; Chan, C. K.; Chan, C.-M. Clean Graphene Surface through High Temperature Annealing. *Carbon N. Y.* **2015**, *94*, 740–748. https://doi.org/10.1016/J.CARBON.2015.07.046.

(6) Choi, W. J.; Chung, Y. J.; Park, S.; Yang, C.-S.; Lee, Y. K.; An, K.-S.; Lee, Y.-S.; Lee, J.-O. A Simple Method for Cleaning Graphene Surfaces with an Electrostatic Force. *Adv. Mater.* **2014**, *26* (4), 637–644. https://doi.org/doi:10.1002/adma.201303199.

(7) Da-Cheng, M.; Song-Ang, P.; Shao-Qing, W.; Da-Yong, Z.; Jing-Yuan, S.; Xinnan, W.; Zhi, J. Towards a Cleaner Graphene Surface in Graphene Field Effect Transistor via N,N-Dimethylacetamide. *Mater. Res. Express* **2016**, *3* (9), 95011.

(8) Tyagi, A.; Mišeikis, V.; Martini, L.; Forti, S.; Mishra, N.; Gebeyehu, Z. M.; Giambra, M. A.; Zribi, J.; Frégnaux, M.; Aureau, D.; Romagnoli, M.; Beltram, F.; Coletti, C. Ultra-Clean High-Mobility Graphene on Technologically Relevant Substrates. *Nanoscale* **2022**, *14* (6), 2167–2176. https://doi.org/10.1039/d1nr05904a.

(9) Fischer, L. M.; Tenje, M.; Heiskanen, A. R.; Masuda, N.; Castillo, J.; Bentien, A.; Émneus, J.; Jakobsen, M. H.; Boisen, A. Gold Cleaning Methods for Electrochemical Detection Applications. *Microelectron. Eng.* **2009**, *86* (4–6), 1282–1285. https://doi.org/10.1016/j.mee.2008.11.045.

(10) Thodkar, K.; Thompson, D.; Lüönd, F.; Moser, L.; Overney, F.; Marot, L.; Schönenberger, C.; Jeanneret, B.; Calame, M. Restoring the Electrical Properties of CVD Graphene via Physisorption of Molecular Adsorbates. *ACS Appl. Mater. Interfaces* **2017**, *9* (29), 25014–25022. https://doi.org/10.1021/acsami.7b05143.

(11) Miller-Chou, B. A.; Koenig, J. L. A Review of Polymer Dissolution. *Prog. Polym. Sci.* **2003**, *28* (8), 1223–1270. https://doi.org/https://doi.org/10.1016/S0079-6700(03)00045-5.

(12) Papanu, J. S.; Hess, D. W.; Soane (Soong), D. S.; Bell, A. T. Swelling of Poly(Methyl Methacrylate) Thin Films in Low Molecular Weight Alcohols. *J. Appl. Polym. Sci.* **1990**, *39* (4), 803–823. https://doi.org/10.1002/app.1990.070390404.
